# The Effects of Ascorbate, *N*-Acetylcysteine, and Resveratrol on Fibroblasts from Patients with Mitochondrial Disorders

**DOI:** 10.3390/jcm6010001

**Published:** 2016-12-22

**Authors:** Liza Douiev, Devorah Soiferman, Corinne Alban, Ann Saada

**Affiliations:** Monique and Jacques Roboh Department of Genetic Research and the Department of Genetic and Metabolic Diseases, Hadassah-Hebrew University Hospital, 91120 Jerusalem, Israel; liza.douiev@mail.huji.ac.il (L.D.); devorah228@gmail.com (D.S.); Korin@hadassah.org.il (C.A.)

**Keywords:** mitochondrial disease, fibroblast, ascorbate, *N*-acetylcysteine, resveratrol, ATP, ROS, OXPHOS

## Abstract

Reactive oxygen species (ROS) are assumed to be implicated in the pathogenesis of inborn mitochondrial diseases affecting oxidative phosphorylation (OXPHOS). In the current study, we characterized the effects of three small molecules with antioxidant properties (*N*-acetylcysteine, ascorbate, and resveratrol) on ROS production and several OXPHOS parameters (growth in glucose free medium, ATP production, mitochondrial content and membrane potential (MMP)), in primary fibroblasts derived from seven patients with different molecularly defined and undefined mitochondrial diseases. *N*-acetylcysteine appeared to be the most beneficial compound, reducing ROS while increasing growth and ATP production in some patients’ cells. Ascorbate showed a variable positive or negative effect on ROS, ATP production, and mitochondrial content, while incubation with resveratrol disclosed either no effect or detrimental effect on ATP production and MMP in some cells. The individual responses highlight the importance of investigating multiple parameters in addition to ROS to obtain a more balanced view of the overall effect on OXPHOS when evaluating antioxidant treatment options for mitochondrial diseases.

## 1. Introduction

Mitochondria are double-membrane-bound organelles with a separate genome, present in nucleated eukaryotic cells. They are involved in several cellular processes and functions, including the crucial generation of cellular energy in the form of adenosine triphosphate (ATP) by oxidative phosphorylation (OXPHOS). This system is composed of ~90 proteins organized into five multimeric protein complexes, coenzyme Q_10_ (CoQ_10_), and cytochrome *c*. Electrons derived from the Krebs cycle are transported along the mitochondrial respiratory chain (MRC) complexes I–IV, resulting in the formation of an electrochemical proton gradient across the mitochondrial inner membrane, which is subsequently utilized by complex V, ATP synthase, to generate ATP [1].

Mitochondrial diseases are a group of inherited diseases of clinical and genetic heterogeneity. The estimated prevalence of adult mitochondrial diseases is approximately 1:4300 [2], while the minimum birth prevalence of mitochondrial respiratory chain disorders is approximately 6.2:100,000 [3]. Mitochondrial diseases are characterized by reduced OXPHOS activity; they may present at any age and may affect a wide range of tissues. The underlying molecular defect may arise either from maternally inherited mutations in the mitochondrial genome (mtDNA) or from mutations in the nuclear genome. Disorders affecting a single complex are caused by mutations in either 1 of the 13 mtDNA-encoded MRC subunits (complexes I, III–V) or one of their nuclear-encoded subunits or assembly factors (complex II is entirely nuclear-encoded). Diseases affecting multiple complexes are usually caused by mutations in genes affecting mtDNA translation and maintenance encoded in the mitochondria (mt-RNA or mr-RNA) or in one of the many nuclear-encoded mitochondrial translation and replication factors [4]. OXPHOS dysfunction may also be secondary to defects in mitochondrial dynamics (fusion and fission), lipid milieu, or cofactor biosynthesis [1,5,6].

A main consequence of OXPHOS dysfunction, in addition to ATP depletion, is the increased generation of reactive oxygen species (ROS). Normally, approximately 2% of the total oxygen consumed by mitochondria is converted into ROS as a byproduct of the MRC. A certain level of ROS production is vital to normal cellular function, as they play a pivotal role in cell signaling [7]. However, when the electron transport is obstructed [8,9], excessive ROS production occurs, with consequent oxidative stress and cell damage. Therefore, it is crucial that ROS levels are tightly regulated.

Although advances have been made in our technical abilities to diagnose and identify the genetic causes of mitochondrial diseases, the treatment possibilities are mostly limited to preventing the progression of the disease or treating symptoms or complications. Over the years, several approaches have been proposed and investigated. One of these is the administration of small molecules to improve mitochondrial functions such as mitochondrial biogenesis, the efficiency of the respiratory chain and the production of ATP, and scavenging of excess ROS [10]. These molecules may be divided into several groups according to their mode of action within the cells. One important group is the antioxidants. In the current study, we aimed to evaluate the effects of three antioxidants on various mitochondrial parameters using patients’ fibroblasts as a model system, employing a platform that we have previously described [11].

The first compound that was assessed is ascorbate (vitamin C, ASC). One of ascorbate’s vital functions is that of a ROS scavenger, due to its ability to be oxidized into two forms of radicals, semidehydroascorbate (SDA) and dehydroascorbate (DHA). These two forms are much more stable and less reactive than free radicals [12]. Moreover, ascorbate has been shown to protect against lipid peroxidation of the cell membrane in a process that involves the regeneration of tocopherol (vitamin E) [13].

The second compound was *N*-acetylcysteine (NAC), which is an acetyl derivate of l-cysteine containing a thiol group. *N*-acetylcysteine can be oxidized by a wide variety of radicals, like most molecules that have thiol groups. *N*-acetylcysteine is known to be involved in several biochemical pathways. One of the main mechanisms is its function as a precursor of cysteine, which is the rate-limiting component of glutathione (GSH), an important antioxidant that is involved in numerous physiological processes. Moreover, *N*-acetylcysteine by itself can serve as an antioxidant by reacting directly with free radicals [14,15].

The third compound assessed was resveratrol (3,5,4-trihydroxylstilbene, RSV). Resveratrol is a polyphenol and phytoalexin found in a variety of plant species, where it is used as a defense mechanism against environmental stress. Some studies reported resveratrol to function as a mitochondrial biogenesis-inducing factor in several model systems. Resveratrol was also reported as a factor that upregulates the expression of several cellular antioxidants and concomitantly downregulates the expression of the ROS-forming NADPH oxidase type 4 (NOX4) [16]. Several in vitro and in vivo studies over recent years have shown that resveratrol has some antioxidative and anti-inflammatory characteristics [17,18].

We evaluated the effects of the above compounds not only on ROS production but also on several OXPHOS parameters in order to obtain a more comprehensive view of their effect on normal and patients’ cells

## 2. Experimental Section

### 2.1. Materials

Cell culture media and solutions were from Biological Industries (Kibbutz Beit Ha’emek, Israel).

Fluorescent mitochondrial dyes were from Invitrogen (Carlsbad, CA, USA), H_2_DCFDA from Biotium (Hayward, CA, USA), and the ATPlite™ was from (PerkinElmer, Waltham, MA, USA). Other reagents were purchased from Sigma-Aldrich Israel Ltd. (Rehovot, Israel) at the highest purity available.

### 2.2. Tissue Culture

Previously established primary skin fibroblast cell lines passage 3–4 (stored in liquid nitrogen) available for the study were derived from patients and controls with informed consent and IRB approval. Cells were cultured in a permissive high-glucose–DMEM (GLU) medium containing 4.5 g/L glucose supplemented with 15% fetal calf serum (FCS), 1% penicillin–streptomycin, 365 μg/mL l-glutamine, 110 μg/mL pyruvate, and 50 μg/mL uridine at 37 °C in 5% CO_2_

Evaluation of compounds was carried out essentially as we previously described [11] as follows: 3000 cells per well were seeded in triplicate on identical 96-well microtiter plates. The following day, the medium was replaced, after rinsing once with phosphate-buffered saline (PBS), with fresh medium or with restrictive medium (GAL: glucose-free DMEM supplemented with 10% dialyzed fetal calf serum, 1% penicillin–streptomycin, 365 μg/mL l-glutamine, and 5 mM galactose) with or without additional compounds as follows: 12 μM ascorbate (ASC, prepared fresh in growth medium), 1 mM *N*-acetylcysteine (NAC, prepared fresh in growth medium), or 25 μM resveratrol (RSV, 200 mM stock solution in DMSO stored at −80 °C). Cells were incubated for 72 h at 37 °C in 5% CO_2_.

### 2.3. Assays in Microtiter Wells

Cell growth was estimated after 72 h by measuring cell content by a colorimetric method using methylene blue (MB) as we have previously described [11]. Absorbance was measured at 620 nm. Intracellular ROS production was monitored by H_2_DCFDA as we have previously described [11], and normalized to cell content. ATP production was measured in digitonin-permeabilized cells in the presence of glutamate and malate essentially as was previously described [19], but modified for adherent cells in microtiter wells as follows; the growth medium was removed, and the wells were rinsed with assay buffer (25 mM Tris pH7.4, 10 mM potassium phosphate pH 7.4, 150 mM KCl, 0.25 mM bovine serum albumin-fatty acid free, digitonin 40 μg/mL). Assay buffer (50 μL) containing 5 mM glutamate, 1 mM malate, and 1 mM ADP were added per well, and the plate was incubated for 30 min at 37 °C. Subsequently, ATP was measured by luciferin–luciferase using the ATPlite™ (PerkinElmer, Waltham, MA, USA) luminescence assay system according to the manufacturer’s instructions.

Mitochondrial content and mitochondrial membrane potential (MMP) were measured by MitoTracker Green FM (MTG) and tetramethylrhodamine ethyl ester (TMRE) (Molecular Probes, Eugene, OR, USA), respectively, as we have previously described [11].

All microtiter plate measurements were performed with a Synergy HT microplate reader instrument (BioTek, Winooski, VT, USA).

### 2.4. Respiratory Chain Enzymes

Citrate synthase, rotenone sensitive NADH coenzyme Q reductase (complex I), succinate dehydrogenase (complex II), succinate cytochrome *c* reductase (complex II + III), and cytochrome *c* oxidase (complex IV, COX) were determined in isolated fibroblast and muscle mitochondria using standard spectrophotometric methods [20,21]. Results were normalized to the activity of the mitochondrial matrix enzyme citrate synthase and expressed as percentage residual activity compared to normal controls (*n* = 10).

### 2.5. Statistical Analysis

Data are expressed as mean ± SEM and were analyzed using the two-tailed Student’s *t*-test and one-way ANOVA using SPSS version 20. *p* < 0.05 was regarded as statistically significant.

## 3. Results

### 3.1. Mitochondrial Respiratory Chain Enzymes

The current study included three control and seven patients’ primary fibroblast cell lines which were divided into three groups according to the type of OXPHOS deficiency. The first group consists of two patients with combined respiratory chain deficiency caused by mutations in nuclear-encoded components of the mitochondrial translation system (the mitochondrial elongation factor *EFTs* [22] and the mitochondrial ribosomal protein *MRPS22* [23]). The second group contains three patients who displayed an isolated OXPHOS defect; one patient has an undefined mutation and displayed a partial NADH coenzyme Q reductase (complex I) deficiency, and two patients are characterized by deficient cytochrome *c* oxidase (complex IV) activity, in one instance due to an undefined mutation and in the other, a missense mutation in the nuclear *COX6B1* gene [24]. The third category includes indirect OXPHOS defects, one patient with lipoamide dehydrogenase (pyruvate dehydrogenase-subunit E3, PDH-E3) deficiency [25], and the other harboring a heterozygous dominant mutation in the *DNM1L* gene, which encodes one of the major mitochondrial fission proteins, the dynamin related protein 1 (DRP1) [26].

The clinical details of the five molecularly defined patients have been previously published. To ensure that the phenotype is preserved in the fibroblasts, we measured the relevant enzymatic respiratory chain activities in fibroblasts (Table 1). Data available from four patients that underwent muscle biopsies are included for comparison.

### 3.2. ROS Production

**Figure 1:**
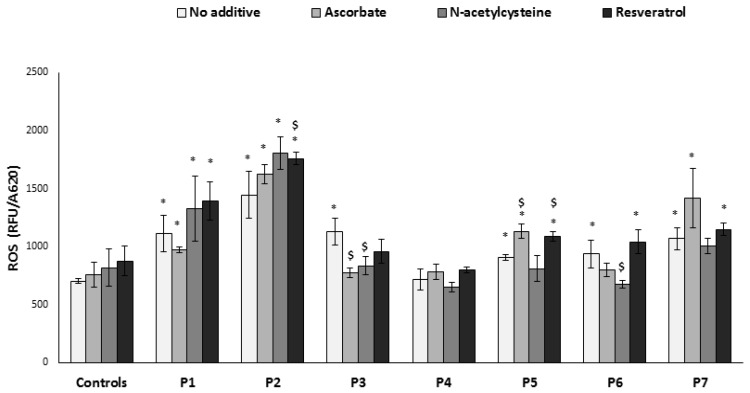
Reactive oxygen species (ROS) production. Fibroblasts in microtiter wells were incubated in the absence or presence of the compounds for 72 h, ROS production (relative fluorescence units, RFU) was determined and normalized to cell content (A620). Results are presented as mean ± SEM of triplicate wells of at least two independent experiments. * *p* < 0.05 compared to mean of 3 controls in the corresponding medium, $ *p* < 0.05 compared to individual patient cells without additive.

Using the microtiter well system (Experimental Section 2.2 and Section 2.3) we studied the impact of the different OXPHOS defects on the intracellular ROS production. All cells, with the exception of P4, showed significantly elevated ROS compared to control cells (*n* = 3) (Figure 1). This result provided the basis for the subsequent evaluation of the effects of three antioxidants (ascorbate, *N*-acetylcysteine, and resveratrol).

Antioxidant concentrations (see Experimental Section 2.2) were chosen according to our preliminary experiments (not shown) and the literature. Fibroblasts were grown for 72 h in the presence of the compounds and washed prior to the determination of ROS production. There was no significant effect on control cells and, contrary to expectation, ROS was not decreased in the patients’ cells with the most elevated initial ROS production (P1 and P2). Moreover, resveratrol significantly elevated ROS in P2. This was also the case for P5 with ascorbate and resveratrol. In fact, only two cell lines with intermediate initial ROS production benefitted from *N*-acetylcysteine (P3 and P6) and ascorbate (P3). Thus, it seems that the common denominators for positive effect are both the nature of the defect or the mutation and the initial basal, intermediate ROS production.

### 3.3. Growth on Glucose-Free Medium

**Figure 2:**
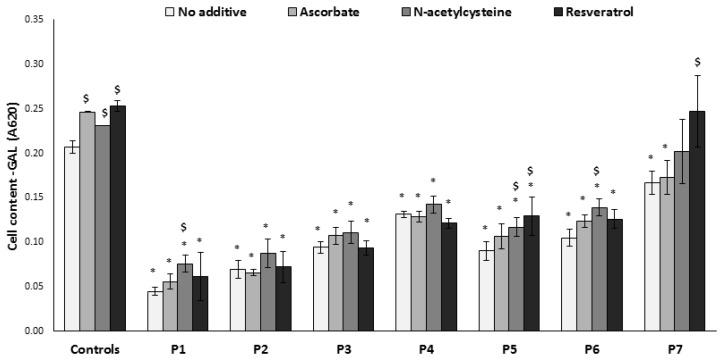
Growth in glucose-free medium. Equal numbers of fibroblasts were seeded in microtiter wells in glucose-free (GAL) medium in the absence or presence of the compounds for 72 h. Growth was determined by measuring cell content by methylene blue (A620). Results are presented as mean ± SEM of triplicate wells of at least two independent experiments. * *p* < 0.05 compared to mean of 3 controls in the corresponding medium, $ *p* < 0.05 compared to individual patient cells without additive.

Cells with impaired OXPHOS generally display markedly decreased cell growth in restrictive conditions (i.e., when grown in medium lacking glucose, in which cells are forced to produce energy via OXPHOS mainly fueled by glutamate and fatty acids). This was also the case for the cells included in this study, reflecting their phenotype (Figure 2). Thus, this feature is a valuable tool for studying the impact of compounds on mitochondrial function. Accordingly, we added this parameter to evaluate the effects of the abovementioned antioxidants. Growth of normal cells is also challenged by this condition; interestingly the controls benefitted slightly but significantly from supplementation with all compounds tested. *N*-acetylcysteine showed a positive trend on all cells, with a statistically significant effect in three patients (P1, P5, P6). Resveratrol significantly improved growth in two cells (P5, P7), while ascorbate had no significant positive or negative effect on patients’ cells. Notably, in terms of growth, the cell with the most severe enzymatic defect (P5, undetectable complex IV activity) significantly benefitted from two antioxidants.

### 3.4. ATP Production

**Figure 3:**
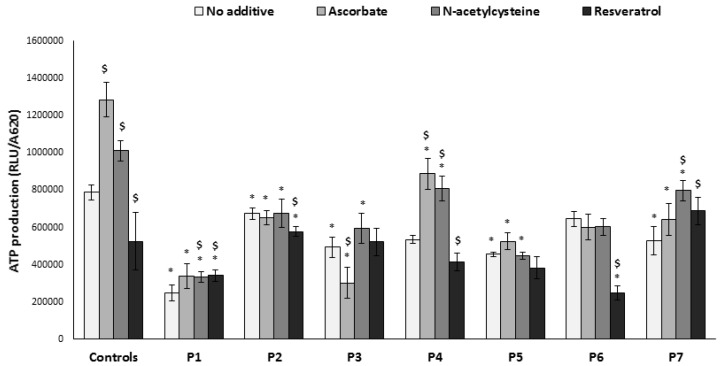
ATP production. Cells were grown in the absence or presence of the compounds for 72 h in microtiter wells. Subsequently, wells were washed, permeabilized, and incubated with glutamate and malate in the presence of ADP; ATP produced was analyzed by luciferin–luciferase (relative luminescence units, RLU). Results were normalized to cell content measured in parallel wells (A620). Results are presented as mean ± SEM of triplicate wells of at least two independent experiments. * *p* < 0.05 compared to mean of 3 controls in the corresponding medium, $ *p* < 0.05 compared to individual patient cells without additive.

ATP production by OXPHOS is the main cellular function of the mitochondria. Accordingly, ATP production from glutamate was significantly lower in all fibroblasts with a defect affecting the MRC compared to controls (Figure 3). ATP production in P6 was slightly but not significantly decreased, possibly due to the fact that the primary disorder is a Krebs cycle defect. Among the compounds tested, *N*-acetylcysteine was the most favorable, increasing ATP production in controls as well as in P4 and P7. Ascorbate had a mixed effect: while favorable in controls, P1, and P4, the compound decreased ATP in P3, who has an undefined defect affecting complex I. Likewise, a mixed response was exerted by resveratrol, which significantly decreased ATP production in controls, P2, P4, and P6, yet slightly increased ATP production in P1 and P7.

### 3.5. Mitochondrial Content

**Figure 4:**
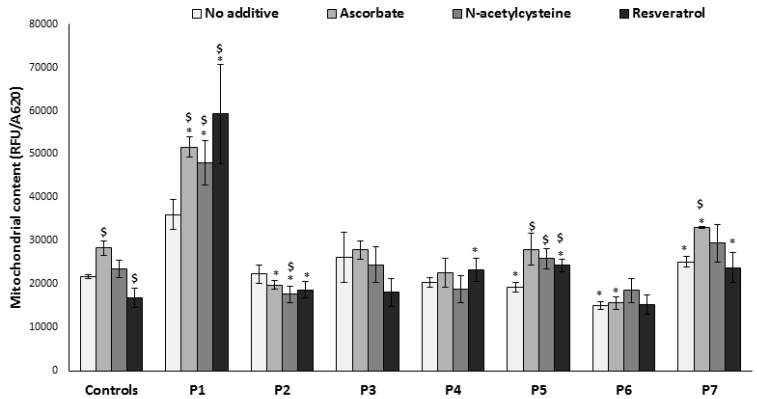
Mitochondrial content. Cells were grown in the absence or presence of the compounds for 72 h in microtiter wells, and stained live with MitoTracker Green (RFU). Results were normalized to cell content measured in parallel wells (A620). Results are presented as mean ± SEM of triplicate wells of at least two independent experiments. * *p* < 0.05 compared to mean of 3 controls in the corresponding medium, $ *p* < 0.05 compared to individual patient cells without additive.

In order to investigate the possibility that variations in ROS and ATP might be due to variable mitochondrial content, we measured mitochondrial mass by live stain with MitoTracker Green, a fluorescent dye localizing to mitochondria independent of the mitochondrial membrane potential (Figure 4). The initial mitochondrial content in most patients’ cells was similar or only slightly different from controls, thus significant differences in ROS production and ATP could not be attributed to mitochondrial content. An exception was found in P1 cells, which displayed a markedly elevated mitochondrial content that could possibly be a compensatory mechanism. However, decreased ATP production (Figure 1) and high ROS (Figure 3) show that this mechanism was probably not very efficient. P6 had lower mitochondrial mass compared to all the other cells, a possible attempt to decrease ROS while managing to maintain an efficient ATP production (Figure 3). The effect of additives was variable and somewhat unpredictable; for example, resveratrol significantly decreased mitochondrial content in controls, but increased in two patients’ cells (P1 and P5). There was no concomitant increase of ATP production in P5 (Figure 3). Ascorbate increased mitochondrial content in controls and in three patients’ cells (P1, P5, P7) with no or positive effect on ATP production (Figure 3) and growth (Figure 2), although the elevated mitochondrial content probably contributed to increased ROS in P5 and P7. *N*-acetylcysteine increased mitochondrial content in three patients’ cells (P1, P5, P7), while increasing ATP production in P1 and P7 (Figure 3) and improving growth in P1 and P2 (Figure 2). Increased mitochondrial content induced by *N*-acetylcysteine occurred without elevating ROS, making it an overall positive compound in the cells tested.

### 3.6. Mitochondrial Membrane Potential

**Figure 5:**
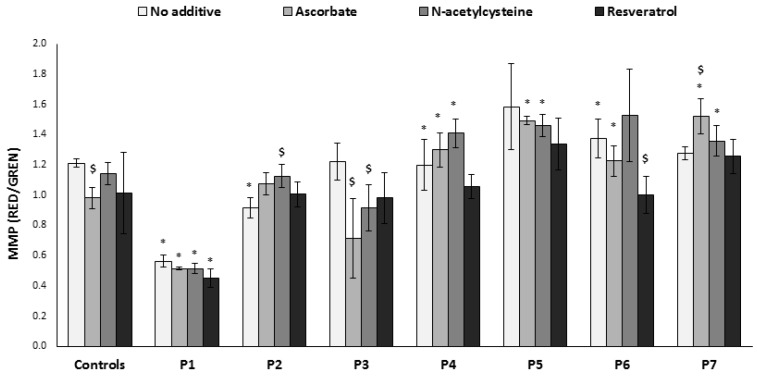
Mitochondrial membrane potential (MMP). Cells were grown in the absence or presence of the compounds for 72 h in microtiter wells, and stained live with tetramethylrhodamine ethyl ester (TMRE) (RED) and MitoTracker Green (GREEN). Results are presented as RED/GREEN ratio mean ± SEM of triplicate wells of at least two independent experiments. * *p* < 0.05 compared to mean of 3 controls in the corresponding medium, $ *p* < 0.05 compared to individual patient cells without additive.

Oxidative stress damage, uncoupling, and/or ATP depletion cause decreased mitochondrial membrane potential (MMP), while a blocked MRC could lead to “hyperpolarization”, both with subsequent detrimental effects on cellular homeostasis. Thus, maintaining the MMP within normal limits is a prerequisite for cell function. Therefore, the MitoTracker Green staining was combined with an MMP-sensitive dye, TMRE, to specifically assess MMP per mitochondrial mass, independently of cell growth (Figure 5). With the exception of P1 fibroblasts, the MMP was slightly (but not markedly) altered in the other patients’ cells compared to controls. In P2, the slightly reduced MMP was improved by *N*-acetylcysteine, while the same compound apparently reduced MMP in P3 but to a level not significantly different from controls. Ascorbate also decreased MMP in P3, concomitant with a marked negative effect on ATP production (Figure 3), although ROS levels were lowered (Figure 1). Resveratrol showed a tendency to decrease MMP (which was significant in P6 and most probably was the cause for decreased ATP production), while ROS remained elevated, making this an overall negative compound for these cells.

## 4. Discussion

The reported prevalence of mitochondrial disorders is steadily increasing, mostly due to the availability of novel diagnostic tools such as exome sequencing. Still, biochemical investigation is usually required in order to complete the verification and characterization of new genetic variants, when molecular diagnosis cannot be reached. Despite major advances in the ability to diagnose and characterize mitochondrial disorders, treatment remains a challenge. Several therapeutic approaches have been proposed and investigated in the past years with limited outcome. Pharmacological treatment options of mitochondrial disorders may include vitamins and cofactors, inducers of mitochondrial biogenesis, and compounds aimed at increasing mitochondrial electron flux and, ultimately, ATP production [10,27]. In patients with MRC dysfunction, inefficient electron transfer often results in increased ROS production. As expected, cells in our study which were derived from patients with different disorders/mutations displayed individual responses to the different compounds, highlighting the possibility to perform compound-screening on a personalized basis. Despite the important physiological role of ROS in signaling, high levels of these noxious molecules may cause oxidative damage to mitochondrial DNA, proteins, and lipids, leading to a vicious cycle of ROS production [28,29]. The observation of severe phenotypes in patients whose relatively mild MRC enzymatic defect would not be expected to significantly decrease ATP production highlights the importance of increased ROS production as an underlying cause of mitochondrial disease. Indeed, increased superoxide formation preceded impaired cellular metabolism following MRC inhibition in an astrocyte model [30]. This was also exemplified in the present study: ROS was significantly elevated, while ATP production was only slightly affected in P2 fibroblasts. Accordingly, it is logical that many therapies for mitochondrial disorders are based on the use of antioxidants to ameliorate the effects of excessive ROS [27,31]. For example, a four-year follow-up of Friedriech ataxia patients treated with combined CoQ10 and vitamin E showed sustained improvement in mitochondrial energy synthesis, while disease progression slowed slightly and cardiac function improved [32]. Additionally, the drug EPI-743 showed initial positive results in children with Leigh syndrome and additional trials are in progress [33]. Furthermore, several of the traditional antioxidants are undergoing modification in order to improve their mitochondrial targeting and clinical efficacy [34], and it is hoped that these compounds will have a positive contribution to the field of mitochondrial disorders therapeutics. Still, due to the heterogeneity of mitochondrial disorders and their variable impact on OXPHOS and cellular metabolism, there is a limited ability to systematically evaluate the outcomes of various treatments. In this study, we evaluated the effects of three antioxidants (*N*-acetylcysteine, ascorbate, and resveratrol) that were suggested in the literature as being effective ROS scavengers. We aimed to systematically evaluate these compounds on normal and mitochondrial patients’ fibroblasts by measuring multiple parameters linked to mitochondrial function. The study was conducted in microtiter plates, as primary fibroblasts grow slowly with a limited passage number. We opted for these cells as they more accurately reflect the defect than transformed cells that tend to lose the original phenotype (AS, personal experience).

Among the tested compounds, *N*-acetylcysteine appeared to be the most beneficial compound, reducing ROS while increasing growth and ATP production in some patients’ cells without an apparent negative effect on MMP. Thus, *N*-acetylcysteine, approved and commonly used in the treatment of paracetamol overdose by maintaining reduced glutathione levels, could possibly be of benefit for certain mitochondrial disorders [14,35]. Moreover, *N*-acetylcysteine has been shown to attenuate oxidative stress, induce cell survival, reverse mitochondrial depolarization, and increase the activities of complexes I, IV, and V in a variety of models and cell lines [36,37,38,39].

Ascorbate plays an important role in biological systems, as both ascorbate [40] and the ascorbyl radical can react directly with many oxidants. Ascorbate can also participate in the regeneration of tocopherol, which can protect against lipid peroxidation of the cell membrane [13]. Previous studies in fibroblasts derived from patients with mitochondrial disorders reported that ascorbate significantly decreased the amount of superoxide radicals and concomitantly increased the activities of complex I–III and II–III of the MRC [40]. Higher ascorbate concentrations were able to partially or totally restore activities of complexes IV, I–III, and II–III in aged (high passage number) fibroblasts [41].

In our current study, ascorbate exerted a variable, patient-specific effect on both ROS and ATP production. This was exemplified by its effect on fibroblasts with impaired complex I activity (P3), where ascorbate supplementation significantly decreased ROS but negatively affected ATP production, possibly due to an excess of reducing power. On the other hand, ascorbate significantly increased mitochondrial content in controls and some patient cells lines, which may explain the elevated ROS and ATP production in these cells, in accord with our previously reported results [24]. Unexpectedly, ascorbate increased ROS production in two of the patients’ fibroblasts, appearing to act as a prooxidant; however, this could in part be explained by the increased mitochondrial content.

The third compound tested was resveratrol, a naturally occurring polyphenol which, apart from its antioxidant properties, can activate AMP-activated protein kinase (AMPK) in a sirtuin (SIRT)1-dependent manner, increasing transcription of nuclear genes involved in energy metabolism. This leads to mitochondrial biogenesis and to upregulation of antioxidant defense systems, thus attenuating mitochondrial ROS production [42]. Experiments performed in fibroblasts of patients with MRC enzyme deficiencies have shown upregulation of expression and activity of MRC enzymes via estrogen receptor and estrogen-related receptor alpha signaling, and correction of moderate complex I deficiencies accompanied by attenuation of oxidative stress mediated by superoxide dismutase 2 (SOD2) and SIRT3 [43,44].

In line with resveratrol’s role as an inducer of mitochondrial biogenesis, a significant increase in mitochondrial content was observed in two patient cells, which may partially explain their increased ROS, while this did not occur in normal cells. In the current study, incubation with resveratrol led to mixed, mostly detrimental, responses on ATP production and MMP. Notably, the limitations of this study are the relatively small sample sizes and that the data were obtained from one dose at one time point. This is due to the fact that primary fibroblasts are restricted in supply and growth. On the other hand, primary cells are preferable because they retain their phenotype, which more accurately reflects the patient’s condition than transformed cell lines. Still, the present finding are in accord with our previous findings for complex I deficiency, and with those of others who reported mixed negative effects of resveratrol [11,45]. Indeed, attempts are being made to harness the cytotoxic effects of resveratrol in the development of cancer treatments [46].

## 5. Conclusions

Generally, patients’ fibroblasts responded differently to the antioxidants, giving further support to our hypothesis that treatments for mitochondrial disorders should be evaluated on a personal basis, depending on the individual underlying genetic defect as well as the genetic background. The individual responses highlight the importance of investigating multiple parameters, and not only ROS, to obtain a more balanced view of the overall effect on OXPHOS when evaluating antioxidant treatment options for mitochondrial diseases.

## Figures and Tables

**Table 1:** Mitochondrial enzymatic activities in patients’ fibroblasts.

Fibroblast	Defect	Activity *		Mutation
		Fibroblasts	Muscle	
**P1**	Combined defect	CI 44% CIV 25%	CI 12%	EFTs^R333W/R333W^
			CII + III 17%	
			CIV 14%	
**P2**		CI 51% CIV 65%	CI 42%	MRPS22^R170H/R170H^
			CII + III 12%	
			CIV 8%	
**P3**	Single defect	CI 44%	-	*undefined*
**P4**		CIV 43%	-	*undefined*
**P5**		CIV Undetectable	CIV undetectable	COX6B1^R20C/R20C^
**P6**	Indirect defect	Lipoamide dehydrogenase 36%	-	LAD^D479V/D479V^
**P7**		CI 58%	CIV 47%	DNM1L^G362S/+^
		CIV 29%		

* Residual enzymatic activity normalized to citrate synthase and compared to controls. Activities within normal ranges are not shown.
